# Social Network Analysis of Cattle Movement in Sukhothai Province, Thailand: A Study to Improve Control Measurements

**DOI:** 10.1155/2015/587252

**Published:** 2015-12-31

**Authors:** Supot Noopataya, Sukanya Thongratsakul, Chaithep Poolkhet

**Affiliations:** ^1^Department of Veterinary Public Health, Faculty of Veterinary Medicine, Kasetsart University, Kamphaeng Saen Campus, Nakhon Pathom 73140, Thailand; ^2^Department of Livestock Development, Ministry of Agriculture and Cooperatives, Bangkok 10400, Thailand

## Abstract

The aim of this study is to analyse the pattern of cattle movement in Sukhothai province, Thailand. A validated questionnaire was applied to 308 respondents related to cattle farming using one-step snowball sampling. The results showed that most of the nodes are farmers who move their animals in the province. The average normalized degree centrality and normalized closeness centrality were low (<0.01 and 0.04, resp.). We found that traders are the nodes with a high value of centrality. This corresponds with the cutpoint analysis results that traders are outstanding. In conclusion, the relevant authorities should focus on the nodes such as traders for controlling disease. However, a measure to detect disease in the early stages needs to be implemented.

## 1. Introduction

One of the most important steps for cattle farming is the transport of cattle from place to place. These movements provide possible modes of transmitting infectious disease. For this reason, many countries have taken measures to control disease, such as restricting animal movement in the controlled areas during occurrence of disease [1]. However, these measures may not be taken in controlling the epidemic because of lack of real understanding about the movement patterns of animals [2–4]. Therefore, it would be valuable to understand the movement patterns of cattle in a developing country such as Thailand. The main point is to know the origin and destination of animal movement. This information can help the relevant authorities to improve disease control measures based on the analysis of scientific data. One of the best tools is the responses from Social Network Analysis (SNA).

SNA is a tool which defines the relationship between units of interest. A unit of interest might be an animal, human, or object, either individually or in a group. In terms of SNA, we usually call the unit of interest a “node.” The “tie” is the term used to explain the relationship between one node and another. In this way, SNA uses the adjacency matrix and graph theory for the method of calculation [5]. Many researchers used the SNA to describe the pattern of diseases widely spreading in human and veterinary medicine [6–9]. Interesting work describes the network of cattle movement in Argentina, which relates to disease spreading. This study indicates that the relevant authorities should focus on the cattle moving out from the highlands to other areas [10]. A study in Great Britain found that the auction market plays an important role in Foot-and-Mouth Disease (FMD) outbreaks [11]. Moreover, Natale et al. showed that the live cattle market is the hub for disease spreading in Italy [12]. This result is similar to that found in a study in Denmark [13].

The aim of this study is to analyse the pattern of cattle movement in Thailand. We selected an area of Sukhothai province because this province is one of the places for cattle husbandry displayed at the live cattle market. Cattle husbandry activity in this area could provide answers about how disease is possibly spread. Additionally, Sukhothai is a perfect place for the study of cattle movement because there are slaughterhouses, backyard cattle farms, and commercial cattle farms in the province. The output from this study can be used to improve or design the control measures. In addition, knowledge of this study might be useful for the authorities in developing countries that have a similar pattern of cattle movement, such as countries in the Mekong subregion of Southeast Asia.

## 2. Materials and Methods

### 2.1. Study Framework and Data Collection

A retrospective and longitudinal study was performed using a validated questionnaire. The questionnaire was presented to respondents involved in the cattle movement network of Sukhothai province, Thailand. In this study, we have focused on beef and dairy movements. All questions of this study focused on cattle movements from January 2011 to December 2011. In this study, we define the node as the farmer, trader, collector, the owner of a slaughterhouse (or slaughterman), and the owner or worker of a live cattle market. For the cattle farms and slaughterhouses, we applied the questionnaire to one person from each location. For the remaining locations, we applied the questionnaires to as many people as we could during the date of data collection (more than 30% of the workers in each location). The tie in this study is the cattle movement among the pair of nodes. It is possible that this movement may or may not pass through the live cattle market. Our calculations did not account for cattle that were sent to a new location but did not arrive and were returned to the old location.

We applied the partial network method with one-step snowball sampling [5]. Data collection activities were started at the live cattle market on June 2012. Then, the information from our respondents about the places they got or bought the animal from (origin) and the places they sold the animal to (destination) pointed to the next locations for data collection. In cases where the original places or destination places were located out of the border of Sukhothai province, we did not follow the link for data collection. Two months later, we collected data at the initial place again. The process of data collection was repeated. At this point, the same respondents from the first data collection were excluded from the analysis. We finished our work on March 2013. A sample size was estimated by Yamane's methods [14]. Based on the suggestion of local authorities, we assumed there are 1,000 stakeholders in this network. We set the alpha error and precision at 5%. As a calculation, we needed the responses of more than 286 people in our study, and we successfully collected 308 responses to our work.

### 2.2. Questionnaire Design

The questionnaire used in the study was clarified by epidemiology experts and tested by asking farmers in the study area for any corrections or modifications. The approved questionnaire was discussed with our teams to clarify the questions before starting the data collection. The questionnaire contained both open and closed questions asked to the respondents in face-to-face interviews. The questions aimed to ask the respondents about general information, their role in cattle farming, trade activities, and movement patterns of the animals. The direction of the cattle movement (where the animal moves from and where the animal moves to) and number of animals moved each time were asked in the questionnaire but were excluded from our analysis to avoid analytical bias from respondents.

### 2.3. Data Analysis

A descriptive statistical analysis was used to explain the basic information of respondents related to their cattle activities. Network analysis was calculated based on the undirected binary network using Ucinet6 (Analytical Technologies, USA) as follows [5, 15].

*Degree Centrality*. It is the normalized value accounted for by analysing the number of ties in each node. The node with the high value of these reflects the high number of ties or the channel of node connection.

*Betweenness Centrality*. It is the Freeman normalized value which is considered the shortest path between two nodes. The node with a high value of these indicated a high frequency of animal movement through the node.

*Closeness Centrality*. It is the normalized value which is considered the geodesic distance from one node to all remaining nodes. A node with a high value indicated that it was easy to move animals to the linking node.

*Component*. It is considered a group of nodes connected to one another by at least one tie.

*Cutpoint*. It is a node whose removal causes the disconnected graph (i.e., creating two or more components).

*Clustering Coefficient*. It is calculated from three connected nodes. This results in a group of nodes forming a triangle shape (transitivity) in the network. The network with the highest probability number of clustering coefficient means that many node triangles are present.

*Density*. It reflects the actual ties that are present in the network compared to possible ties, and calculating density gives us the results of the probability number.

An Exponential Random Graph Model (ERGM) was calculated by using StOCNET [16]. These methods examined the network for possible alternative network based on observed uncertainty data. The features, such as transitive triplets (the amount of transitivity) and dyad (the number of pairwise relations between nodes) count, were examined with the 10,000 iterations of the Monte Carlo Simulation.

Mapping display of all nodes in these networks with ties was performed by using ArcGIS 10.2.1 (ESRI, USA). The respondents' nodes were recorded using their geographical coordinates from handheld GPS devices (Garmin, USA). For nodes unable to have their geographical coordinates located, their position was estimated based on the centroid from the border of subdistricts of Thailand's map using ArcGIS.

## 3. Results

### 3.1. General Information

**Figure 1:**
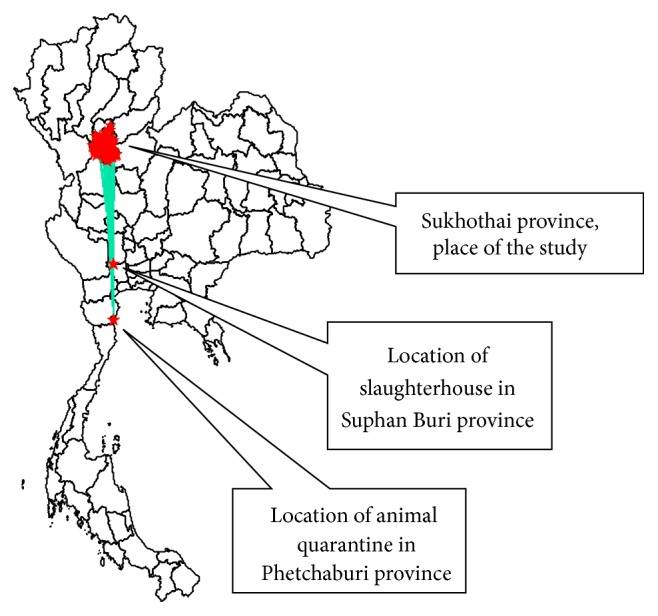
A map of Sukhothai's cattle movement in the year 2012.

According to our interviews with 308 respondents, 224 of them were animal owners, 82 were slaughterhouse staff, 1 was an owner of a private animal quarantine station, and 1 was the owner of a live cattle market. We found that the respondents' average age was 52.72 years (SD = 8.32). Most of them (99.67%) graduated with compulsory education from elementary school, and some respondents held higher academic certificates. Most respondents (93.18%) were living in Sukhothai province. Only 6.82% were traders living in other provinces. For movement distances, the median of these activities was 39.60 kilometres (range = 0–550 kilometres). They usually moved their cattle to farms, slaughterhouses, or live cattle markets. For respondents, like the traders, who moved their animals farther distances, the cattle were moved and quarantined at the government quarantine station in Phetchaburi province. Some of the respondents who moved their cattle to a slaughterhouse were located in Suphan Buri province (Figure 1).

The results show that the animals mostly move within the province, and the number of animals moving is within the range of 1–5 heads. In cases of respondents who move their animals longer distances, they might be capable of increasing the number of cattle up to 30, depending on how many cattle they bought.

### 3.2. Network Analysis

The results show that this network has 2,367 nodes with 3,150 ties. We found that most of the nodes have connections with the others through a few links. The nodes were mostly connected to each other by dyad. The mean (and standard deviation) of normalized degree centrality was less than 0.01 (SD = 0.05), and the highest value and the lowest value of these parameters were 0.13 and 0, respectively. The nodes with the highest normalized degree centrality were a farmer and a trader in Sukhothai province. The node with the lowest normalized degree centrality was a farmer in this area in the cattle market who did not have any transactions during the study and moved the cattle back to the original location. In addition, we found that some farmers raised their animals beside the market for transaction activities to occur in the next week. This meant they lived near the market and took care of their animals until the market opened the following week. The analysis of normalized closeness centrality in this network showed that the average was 0.04 (SD < 0.01). The maximum and minimum values were 0.04, and all were from farmers. Moreover, all nodes represented the null value of normalized betweenness centrality.

**Figure 2:**
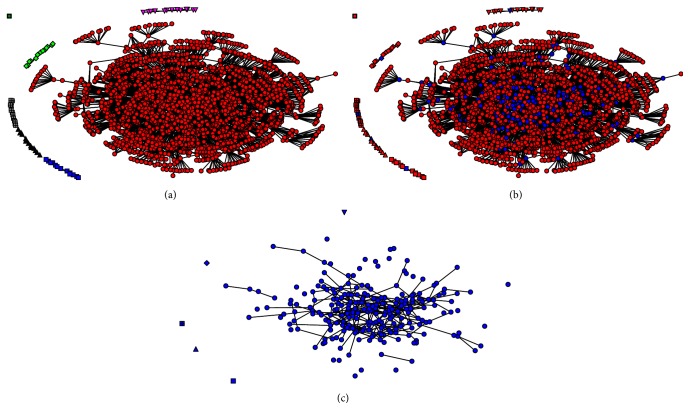
A sociogram of cattle movement in Sukhothai province during 2011. (a) represents the components separated by colour and shape. (b) represents all nodes in the network which were ordinary nodes (red) and cutpoint (blue). (c) only presents cutpoints showing their connections.

The analysis of the network component found that this network has seven components (Figure 2(a)). A main component contained most of the nodes and was located as a core network when multidimensional scaling was used for analysing network representation. This means that most of the nodes were similar. The five remaining components were small and disconnected from each other. We found that one node was identified as an isolator (separate node). The analysis found that the nodes in this network were the cutpoints at 1,565 (66.18%) and there were massive links between cutpoints (Figures 2(b) and 2(c)).

The average network density was 0.003 (SD = 0.109), which reflects the notion that the real movement activities in this network were only 0.3 per cent comparing to all possible movements in the theorem. This correlated with the result of centrality in this network, which had a low value. The clustering coefficient of this network was 0.009. The network density and clustering coefficient were low. This showed that this network had a random pattern. A calculation using ERGMs with Monte Carlo Simulation confirms that all possible dyads in this network should be 2,800,564 nodes or significantly represent 99.93% of the maximum value of dyads (*p* < 0.01). This reflects the notion that this network represents a dense social relationship structure like a dyad. It also proves that this network represents a significant triangle relationship (15; *p* < 0.01).

## 4. Discussion

Based on the respondents' answers and our observation, some participants do not pay much attention to farm biosecurity. The relevant authorities should regularly educate the stakeholders about risk factors, measures of biosecurity, measures for disease monitoring or surveillance, and measures for disease control. This is one way to successfully control and prevent disease in developing countries. Therefore, the relevant authorities must be educating them with easy-to-understand media, such as broadcast media.

We found that the farmers mostly move their cattle within the province and take less than 40 km to transport them each time; however, when cattle are moved to another province, the distances reached would be 550 kilometres. This study shows that the respondents do not move their animals to the neighboring province of Sukhothai, because the movement of animals across the border of province must be allowed by the authorities. They may feel not free to obtain approval documents from the authorities. The control measure of restricting animal movement between the provinces is a tool to help the authorities control the occurrence and spread of disease. If diseases such as FMD occur and spread through cattle movement, it would seem that there are dominant local occurrences within the province because of shorter movement distances. This coincides with the previous studies [17], which look at backyard farmer's behaviour. Another work found that most livestock premises in Peru are infected by the local spread of FMD [18].

Likewise, the disease can spread over long distances, and the pattern of disease distribution possibly jumps to provinces which are disconnected from Sukhothai province. From Figure 1, the disease possibly moved to the upper zone of the southern part of Thailand. From this analysis, we show that if the disease is present in countries with disconnecting geographic patterns of the infected areas, then the vehicle or carrier must be considered. To address these problems, the Department of Livestock Development of Thailand imposed the disease control measures to prevent and control animal diseases such as FMD. They have a system named “e-Movement, an electronic documentation for livestock movement approvals” for animal movement recording, which was designed for recording when the stakeholders move their animals between the provinces. The stakeholders must ask the authorities for permission by documentation and then the authorities will electronically record the data. However, they lack a routine analysis of this data, so we suggested that the analysis of network data is necessary. Moreover, the “e-Movement” does not cover the movement within the province and the Thai authorities need to consider that for effective analysis. For example, if the disease occurs in the area, the farmers or traders are free to move infected animals to other areas within the province. We suggested that a system be designed to fulfil these gaps by extending the documentation for animal movement within the province. Thus, in Thailand, restricting animal movement is a key measurement for disease control during the outbreak situation. It is an administrative measurement at the provincial level only. We recommended that the authorities should improve the system by making it available at the district level.

We found that the majority of activities occurred at the farm, because Sukhothai has only one cattle market open once a week and this might not be enough for slaughtering. Therefore, the traders or other relevant players in the cattle market are looking for more animals. Thai authorities should think about this information for disease prevention and control.

In this study, we used the one-step snowball approach for sampling, which reflects the behaviour of the network as well, and we found that there were problems with complete node identification. In other words, some respondents could not remember exactly the name of the person who sold or bought their animal. This affects the identification of connecting nodes for data collection. We corrected these problems by using the demographic data (we collected the data from nodes who live in the same village) instead of name identification. Then, the nodes that the respondents refer to are possibly different from the data collected nodes. This explains why we found many components in this network. However, this sampling problem was less than 10% of the total data. From our experience, we believe that the results might not be any different between a one-step and a more-than-one-step snowball approach, because most stakeholders have a few links of pair's connections. On the other hand, if we carry out a two-step approach, there will be more recall bias or lying.

For centrality value of network analysis, normalized degree centrality is low, which means the node has moved cattle through a few connections. This is consistent for most respondents, who are small stakeholders or backyard farmers who always sell or buy the animals with familiar people. In the network with high degree centrality, it is hard to control the disease spread compared to the network with low degree centrality [5]. In the network with low connection, the probability of detection will be low because not a lot of nodes will be infected. For this reason, active surveillance or education of farmers on the signs of FMD should be done. On the other hand, the standard deviation of degree centrality is low. This indicates that almost all the nodes in this network are homogeneous, and the multidimensional scaling confirmed this. Thus, there is no hub in this network [12]. The normalized closeness centrality in this network is low. This feature reflects the speed of the message flow [15]. Thus, if the disease occurs in the network, the speed of infection does not move rapidly. This is good for authorities to control the disease. The normalized Freeman betweenness centrality in this network is null, which consequently affects a high number of dyad node relationships. Thus, these statistics might not fit for this kind of network. We suggested that the centrality measures in a network representing a few steps, the degree centrality and closeness centrality, might be useful for centrality measurements. Moreover, the nodes as traders have the highest degree of centrality and they play an important role in spreading disease. The relevant authorities should focus on these nodes to control disease in cattle farming and should implement the active surveillance system on them. In this way, the authorities should request that they notify them of any diseases identified in the early stages to prevent further spreading. However, previous studies show different results in Denmark and Great Britain [11, 13]. They found that the animal market plays a key role in the continued spread of infectious disease. However, this depends on the nature of each country. We had expected that the other developing countries would present network's behaviour similar to Thailand.

As a cutpoint analysis, traders were densely connected together. If the disease spreads in the area, it is possible that traders influence the increase of infectious nodes. It seems appropriate that these nodes need to be under surveillance and control; however, these people are always a hidden population and the authorities need to handle them gently. The researchers were determined that an efficient surveillance system needs good cooperation between the local veterinarian and the relevant people such as farmers [19]. In this study, some farmers also played the role of traders. The trader is a key person for disease control. In addition, the result of cutpoint analysis showed that all traders are the cutpoint nodes which indicate that we can break the infectious network by controlling them. With good control of these nodes, the magnitude of disease during an outbreak can be minimized. It is similar to our previous study in Laos and Cambodia [Authors; in preparation for publishing]. The results of clustering coefficient and density are low. We consider this network to be close to a random network, meaning that a node is connected to the others without a regular pattern. This corresponds with the standard deviation of degree centrality in which the hub is not present. Therefore, a connection from node to node could not predict a certain path [11].

Moreover, the ERGMs showed that the relationship in this network is significantly dominated by a dyad. In terms of infectious control, the dyad relationship is easier to control compared to others [5]. In this network, the triangle relationship is significantly present. In fact, triangle relationship is typically more stable than a dyad [15]. As we described above, Thai authorities need to develop an active surveillance system to detect the early stages of infectious disease with restrictions on current control measurements. This will help the authorities improve effective control measurements. The other developing countries can use this knowledge to develop their control measurements effectively. For further investigation, the influence of risky premises, such as shared grazing areas or other locations, needs to be studied.

## 5. Conclusion

The study of cattle movement using the SNA in Sukhothai province found that traders were the key players in this network. We found that the patterns of spreading diseases, such as FMD, are more locally spread than faraway spread. In this study, the results suggested a need to improve control measurements based on these nodes. We recommended that the following control measurements be developed: a database for animal movement within the provinces; an improvement in the quarantine station for lengthy movements; zoning regulations for cattle farming; control of trader activities; and the development of a highly sensitive disease detection system in the early stages. All of our suggestions are based on the results of descriptive statistics, our experiences, and SNA. These will help us to understand the possible patterns of disease spreading and to improve the control measurements for better control in Thailand and other developing countries which have the same cattle farming behaviours. An analysis of the network with a mathematic model such as SIR model needs to be conducted in further studies.

## References

[1] World Health Organisation for Animal Health (2015). *Foot and Mouth Disease Portal*.

[2] Shirley M. D. F., Rushton S. P. (2005). Where diseases and networks collide: lessons to be learnt from a study of the 2001 foot-and-mouth disease epidemic. *Epidemiology and Infection*.

[3] Dubé C., Ribble C., Kelton D., McNab B. (2009). A review of network analysis terminology and its application to foot-and-mouth disease modelling and policy development. *Transboundary and Emerging Diseases*.

[4] Rivas A. L., Fasina F. O., Hammond J. M. (2012). Epidemic protection zones: centred on cases or based on connectivity?. *Transboundary and Emerging Diseases*.

[5] Borgatti S., Everett G., Johnson C. (2013). *Analyzing Social Networks*.

[6] Curtis R., Friedman S. R., Neaigus A., Jose B., Goldstein M., Ildefonso G. (1995). Street-level drug markets: network structure and HIV risk. *Social Networks*.

[7] Bell D. C., Atkinson J. S., Carlson J. W. (1999). Centrality measures for disease transmission networks. *Social Networks*.

[8] Bigras-Poulin M., Thompson R. A., Chriél M., Mortensen S., Greiner M. (2006). Network analysis of Danish cattle industry trade patterns as an evaluation of risk potential for disease spread. *Preventive Veterinary Medicine*.

[9] Ortiz-Pelaez A., Pfeiffer D. U., Soares-Magalhães R. J., Guitian F. J. (2006). Use of social network analysis to characterize the pattern of animal movements in the initial phases of the 2001 foot and mouth disease (FMD) epidemic in the UK. *Preventive Veterinary Medicine*.

[10] Aznar M. N., Stevenson M. A., Zarich L., León E. A. (2011). Analysis of cattle movements in Argentina, 2005. *Preventive Veterinary Medicine*.

[11] Robinson S. E., Christley R. M. (2007). Exploring the role of auction markets in cattle movements within Great Britain. *Preventive Veterinary Medicine*.

[12] Natale F., Giovannini A., Savini L. (2009). Network analysis of Italian cattle trade patterns and evaluation of risks for potential disease spread. *Preventive Veterinary Medicine*.

[13] Mweu M. M., Fournié G., Halasa T., Toft N., Nielsen S. S. (2013). Temporal characterisation of the network of Danish cattle movements and its implication for disease control: 2000–2009. *Preventive Veterinary Medicine*.

[14] Yamane T. (1967). *Elementary Sampling Theory*.

[15] Prell C. (2011). *Social Network Analysis: History, Theory and Methodology*.

[16] Boer P., Huisman M., Snijders B., Steglich C., Wichers Y., Zeggelink H. (2006). *StOCNET: An Open Software System for the Advanced Statistical Analysis of Social Networks. Version 1.7*.

[17] Poolkhet C., Chairatanayuth P., Thongratsakul S. (2013). Social network analysis for assessment of Avian influenza spread and trading patterns of backyard chickens in Nakhon Pathom, Suphan Buri and Ratchaburi, Thailand. *Zoonoses and Public Health*.

[18] Martínez-López B., Ivorra B., Fernández-Carrión E. (2014). A multi-analysis approach for space-time and economic evaluation of risks related with livestock diseases: the example of FMD in Peru. *Preventive Veterinary Medicine*.

[19] Rautureau S., Dufour B., Durand B. (2011). Vulnerability of animal trade networks to the spread of infectious diseases: a methodological approach applied to evaluation and emergency control strategies in Cattle, France, 2005. *Transboundary and Emerging Diseases*.

